# Lifestyle and the hypertensive disorders of pregnancy in nulliparous women in the United States: a secondary data analysis of the nuMom2b

**DOI:** 10.1186/s12884-023-05522-8

**Published:** 2023-03-23

**Authors:** Elizabeth Mollard, Constance Cottrell

**Affiliations:** 1 College of Nursing, University of Nebraska Medical Center, 550 North 19Th St, # 357, Lincoln, NE 68588-0620 USA; 2 Office of Nursing Research and Innovation, Cleveland Clinic, 9500 Euclid Ave/T4, Cleveland, OH 44195 USA

**Keywords:** Hypertension, Pregnancy, Preeclampsia, Lifestyle, Wellness, Sleep, Physical activity, Nutrition

## Abstract

**Background:**

Hypertensive disorders of pregnancy are a leading cause of maternal and fetal morbidity and mortality and a significant risk factor for future cardiovascular disease development in women. This study aimed to explore lifestyle wellness-related variables and how they impact the risk of hypertension in pregnancy.

**Methods:**

This is a secondary analysis of data from the prospective cohort study Nulliparous Pregnancy Outcomes Study: Monitoring Mothers-To-Be (nuMoM2b). Data was collected through questionnaires, clinical evaluations, and medical records review at 8 academic medical centers in the United States. Four study visits were scheduled throughout the participant’s pregnancy (visits one–four): 6^0^–13^6^, 16^0^–21^6^, and 22^0^–29^6^ weeks gestation and birth. A series of statistical modeling and logistical regression were performed using 15 lifestyle variables related to sleep, nutrition, resilience, illness avoidance, and physical activity were selected as predictor variables with an outcome variable of hypertension.

**Results:**

Of 9289 nulliparous participants considered for inclusion in our analyses, 1464 had any HDP during study participation, and 554 participants had complete data available for the study and were included in our final sample. Results were statistically significant at a level of *p* < 0.05. Of the sleep variables, snoring at visit 1 increased the risk of hypertension in pregnancy. Greater vegetable consumption reported at visit one decreased risks of hypertension in pregnancy. Physical activity reported at visit two and visit three were associated with decreased risk of hypertension. Physical activity reported at visit three combined with more hours of sleep each night, or through napping habit reported at visit one decreased hypertension risk. Increased fish oil consumption combined with more hours of sleep at visit one increased odds of hypertension in pregnancy.

**Conclusions:**

Our results support that lifestyle wellness-related variables relating to sleep, physical activity and nutrition affect hypertension in pregnancy. The studied variables and others should be considered in future research and intervention development to reduce hypertension in pregnancy and improve maternal wellness.

## Background

Hypertension is the most common medical complication experienced by pregnant women and is a leading cause of poor maternal and fetal outcomes. Hypertensive disorders of pregnancy (HDP) increase maternal morbidity, such as heart attack and stroke, pregnancy-related mortality, and future cardiovascular disease development [1, 2]. The number of women experiencing HDP has risen rapidly globally, and in the United States have a prevalence rate of over 15%, impacting one in seven hospital births [1, 3, 4].

Predisposing risk factors for HDP are similar to risk factors for hypertension outside of pregnancy [5, 6]. In the US, risks of HDP are higher in nulliparous women, women of advanced maternal age (≥ 35 and further at ≥ 40), who live in low-income areas, are unemployed, have low educational attainment, live in the South or Midwest, are non-Hispanic Black, non-Hispanic American Indian or Alaska Native race, overweight or obese body mass index, or who have underlying health conditions, including diabetes, kidney disease, and autoimmune disorders [3, 7].

Clinical research on HDP focuses heavily on pharmacological prevention and management of HDP and limited research is conducted on lifestyle and wellness behaviors as they relate to HDP during pregnancy [8–10]. Although pharmacological advances for HDP have improved outcomes and reduced rates of adverse events from HDP, lifestyle and wellness remain critical to overall physiological health and blood pressure [11, 12]. In addition, there is growing evidence that the increased prevalence of HDP is related to lifestyle, primarily due to increases in chronic hypertension, which has risen exponentially over the last 40 years [13].

Lifestyle and wellness recommendations focus on increased adoption of positive health-promoting behaviors such as physical activity, good nutrition, adequate sleep, preventative health measures, and improved resilience [14]. While there are recommendations on post HDP lifestyle modification for long term cardiovascular health, there are limited lifestyle wellness-based guidelines for pregnant populations beyond modifying what is recommended for the general public [6, 15]. Despite that most dietary interventions have been shown to improve pregnancy outcomes in research, nutritional pregnancy guidelines often focus on appropriate weight gain and restricting unsafe foods instead of identifying optimal nutrition strategies for pregnancy [16–18]. While exercise has been shown to decrease risks of some forms of HDP, pregnancy physical activity guidelines are similar to general recommendations of 150 min of moderate-intensity aerobic activity per week [19–22]. Even fewer recommendations are available regarding sleep or fostering or developing traits like resilience in pregnancy. As such, most women do not meet guidelines for the general population for dietary intake or physical activity in pregnancy and are unlikely to be counseled on wellness in areas where guidelines are not established [18, 23–25].

Despite an increase in HDP with an underlying etiology related to lifestyle, there is limited published research on lifestyle or wellness and its relationship to HDP in pregnancy. This study aimed to explore lifestyle wellness variables and their association and interaction with risk for HDP in nulliparous women. We hypothesized that protective lifestyle variables, specifically sleep, physical activity, nutrition, resilience, and preventive health, may be related to a lower risk of HDP in nulliparous women. By identifying lifestyle variables that lower the risk for HDP, future low-cost, easy-to-implement lifestyle interventions can be developed to prevent HDP and promote wellness.

## Methods

### Study design

This is a secondary analysis of data from the Nulliparous Pregnancy Outcomes Study: Monitoring Mothers-To-Be (nuMoM2b), a prospective cohort study designed to evaluate contributors to poor birth outcomes (Clinicaltrials.gov identifier NCT01322529). The methods of this study were described in detail previously and will be described here in brief [26]. The parent study population included 10,037 first-time pregnant women at one of eight academic medical systems in seven states in the US (California, Indiana, Illinois, New York, Ohio, Pennsylvania, and Utah) from October 2010 to September 2013. Eligible women had a viable singleton pregnancy confirmed by ultrasound with a gestation less than 14 weeks at enrollment. Participants had never given birth (20 weeks gestation or greater) and were planning to give birth at a participating site. Exclusion criteria were individuals less than 13 years old, women who planned termination, participants with lethal fetal malformations and aneuploidies, donor oocyte pregnancy, history of three or more pregnancy losses, or inability to provide consent. Appropriate institutional review board approval was obtained at each participating site, and informed consent was collected from each participant.

Study information was obtained throughout participants’ pregnancies concurrently with their prenatal care to study pregnancy outcomes, including HDP. Four study visits were scheduled throughout the participant’s pregnancy. Visit one took place in approximately the first trimester (6 weeks- 13 weeks 6 days gestation). Visit 2 took place in approximately the early second trimester of pregnancy (16 weeks through 21 weeks 6 days gestation), Visit 3 took place in the late 2^nd^ or early 3^rd^ trimester (22 weeks through 29 weeks 6 days gestation), and Visit 4 took place at the time of birth. Data was collected through interviews, questionnaires, clinical evaluations, and medical record review.

### Variables

Fifteen variables related to wellness (nutrition, physical activity, resilience, avoidance of illness as preventative health measures, and sleep) were chosen as predictor variables in the model (Table 1). Sixteen other wellness-related variables were considered but removed due to potential issues with multicollinearity.

**Table 1:** Fifteen chosen lifestyle wellness variables and when they were collected

Variable number	Variable Instrument or Question	Visit collected
Nutrition		
1	Daily caloric intake	Visit 1
2	Healthy Eating Index-2010 (HEI) total (overall)	Visit 1
3	Alternative Healthy Eating Index (AHEI) component scores for vegetable intake	Visit 1
4	Dehydroepiandrosterone (DHEA) and eicosapentaenoic acid (EPA) fatty acid intake	Visit 1
Physical Activity		
5	*“During the past four weeks, did you participate in any physical activities or exercises like running, aerobics, gardening, ball games, or walking for exercise”?*	Visit 1
6		Visit 2
7		Visit 3
Sleep		
8	*“How many hours of sleep do you usually get per night (in hours)*?”	Visit 1
9	*“During a usual week, how many times do you nap for 5 minutes or more?”*	Visit 1
10	“*Do you snore?”*	Visit 1
Resilience		
11	“*Are you able to adapt to change?”*	Visit 2
12	*“Do you tend to bounce back after illness or hardship*?”	Visit 2
Illness avoidance (presumed preventative health practices)		
13	“*Have you had any 'flu-like illnesses,' 'really bad colds,' fever, a rash, or any muscle or joint aches since last study visit?”*	Visit 2
14		Visit 3
15		Visit 4

Four nutrition variables were taken from the modified Block 2005 Food Frequency Questionnaire (FFQ) administered at Visit 1 [12]. The FFQ focused on the foods and nutrients consumed in the three months following conception. Of nutrition variables, Healthy Eating Index-2010 (HEI) total (overall) score provided the level that foods aligned with key dietary recommendations, based on the Health and Human Services and the United States Department of Agriculture *Dietary Guidelines for Americans* [13]. The score ranges from 0 to 100, with a higher score reflecting more optimal alignment with key dietary recommendations. The Alternative Healthy Eating Index (AHEI) component scores for vegetable intake, dehydroepiandrosterone (DHEA) and eicosapentaenoic acid (EPA) fatty acid intake, and daily caloric intake were calculated.

The variable used to measure any physical activity in the past month at Visit 1–3 was: “*During the past four weeks, did you participate in any physical activities or exercises like running, aerobics, gardening, ball games, or walking for exercise”?* This variable was derived from the standardized physical activity questions adapted from the Behavior Risk Factor Surveillance System (BRFSS) [27, 28]. Data were available for each of the trimester visits.

Two variables for resilience were extracted from the Connor Davidson Resilience Scale using a 5-point Likert scale ranging from 0 (not true at all) to 4 (true nearly all of the time) with higher scores being indicative of resilience [29]. The questions from the Connor Davidson, collected at visit two included: “*Are you able to adapt to change?”,* and “*Do you tend to bounce back after illness or hardship*?” Sleep questionnaire data were collected at Visit 1 and included three items**.** The first sleep variable used in our analysis was related to overall hours of sleep per night asked as,** “***How many hours of sleep do you usually get per night (in hours)*?” The second variable was about napping habits, asked as, “*During a usual week, how many times do you nap for 5 min or more?”* Finally, a sleep variable assessing whether the individual snores was a simple question, “*do you snore?*”. The illness avoidance via presumed preventative health practices was measured using the variable, “*Have you had any 'flu-like illnesses,' 'really bad colds,' fever, a rash, or any muscle or joint aches since last study visit?”* at visit two, three, and four.

## Data analysis

Statistical analysis was performed using R Statistical Software (version 4.0.3; R Foundation for Statistical Computing, Vienna, Austria). All participants with available data were considered for this analysis (*n* = 9289) which included 1464 participants with HDP. After removing observations with missing values for any of the 15 selected variables, we were left with 554 participants in the remainder of the analysis.

The correlations between these 15 variables were calculated to ensure multicollinearity would not be a problem (Table 2). No high pairwise correlations were observed, as all absolute correlation values were below 0.5. The distribution of each of these variables was also examined via histogram to check for potential outliers. Based on these distributions, no outliers were removed, and no data transformations were utilized. A logistic regression model was then fit to the data with these 15 variables. Yes/No variables were coded numerically with Yes = 1 and No = 0. All other variables were treated as numeric. The outcome variable was the presence of HDP (Yes = 1, 0 = No).

**Table 2:** Correlations among variables

Variable	CAL	HEI	VEG	FA	PAV1	PAV2	PAV3	SLPHR	NAP	SNORE	RES1	RES2	ILLV2	ILLV3	ILLV4
CAL	1.00	-0.32	0.33	0.18	-0.05	-0.09	-0.18	0.11	0.12	0.02	-0.18	-0.13	-0.01	0.00	-0.03
HEI	-0.32	1.00	0.38	0.23	0.22	0.28	0.27	-0.04	-0.13	-0.01	0.26	0.21	-0.04	0.00	0.03
VEG	0.33	0.38	1.00	0.36	0.15	0.15	0.02	0.02	0.05	0.05	0.08	0.11	-0.04	-0.02	-0.07
FA	0.18	0.23	0.36	1.00	0.10	0.12	0.10	-0.05	-0.03	0.02	0.07	0.05	0.01	0.03	0.09
PAV1	-0.05	0.22	0.15	0.10	1.00	0.42	0.36	-0.02	-0.02	-0.06	0.09	0.10	-0.02	-0.03	0.00
PAV2	-0.09	0.28	0.15	0.12	0.42	1.00	0.44	-0.06	-0.02	0.01	0.04	0.16	-0.03	0.00	0.01
PAV3	-0.18	0.27	0.02	0.10	0.36	0.44	1.00	-0.06	-0.03	0.06	0.14	0.17	0.00	-0.02	0.05
SLPHR	0.11	-0.04	0.02	-0.05	-0.02	-0.06	-0.06	1.00	0.03	-0.03	-0.04	-0.04	-0.01	-0.02	-0.04
NAP	0.12	-0.13	0.05	-0.03	-0.02	-0.02	-0.03	0.03	1.00	0.08	-0.07	-0.10	0.00	0.03	0.03
SNORE	0.02	-0.01	0.05	0.02	-0.06	0.01	0.06	-0.03	0.08	1.00	-0.04	0.01	-0.01	0.05	0.02
RES1	-0.18	0.26	0.08	0.07	0.09	0.04	0.14	-0.04	-0.07	-0.04	1.00	0.46	-0.09	-0.02	0.05
RES2	-0.13	0.21	0.11	0.05	0.10	0.16	0.17	-0.04	-0.10	0.01	0.46	1.00	-0.10	-0.07	0.02
ILLV2	-0.01	-0.04	-0.04	0.01	-0.02	-0.03	0.00	-0.01	0.00	-0.01	-0.09	-0.10	1.00	0.11	-0.02
ILLV3	0.00	0.00	-0.02	0.03	-0.03	0.00	-0.02	-0.02	0.03	0.05	-0.02	-0.07	0.11	1.00	0.10
ILLV4	-0.03	0.03	-0.07	0.09	0.00	0.01	0.05	-0.04	0.03	0.02	0.05	0.02	-0.02	0.10	1.00

Variable abbreviations: *CAL* Daily Caloric intake, *HEI* Healthy Eating Index-2010 total (overall), *VEG* Alternative Healthy Eating Index component scores for vegetable intake, *FA* Dehydroepiandrosterone (DHEA) and eicosapentaenoic acid (EPA) fatty acid intake; PAV1 (visit 1), PAV2(visit 2), PAV3(visit 3) for the question:* “During the past four weeks, did you participate in any physical activities or exercises like running, aerobics, gardening, ball games, or walking for exercise”?; *SLPHR:* “How many hours of sleep do you usually get per night (in hours)?”;* NAP*: “During a usual week, how many times do you nap for 5 minutes or more? *SNORE for the question: *“Do you snore?”;* RES1: *“Are you able to adapt to change?”; *RES2:* “Do you tend to bounce back after illness or hardship*?”; ILLV2 (visit 2), ILLV3 (visit 3), ILLV4 (visit 4) for the question: “*Have you had any 'flu-like illnesses,' 'really bad colds,' fever, a rash, or any muscle or joint aches since last study visit?”*

## Results

Five hundred and fifty-four participants had complete data available for the study and were included in our final sample. The initial run indicated that the following interaction terms were significant at a level of 0.05 (shown with p-values): DHEA and EPA intake with overall hours of sleep per night (variables 4:8) (*p* = 0.003); Physical activity in the past month at both visit two and visit three (variables 6:7) (*p* = 0.04); Physical activity in the past month at visit three and overall hours of sleep per night (variables 7:8) (*p* = 0.02), Physical activity in the past month at visit three and napping habit (variables 7:9) (*p* = 0.01). The following main effects that did not appear in a significant interaction but that were also found to be significant at a level of 0.05 were vegetable intake (variable 3) (*p* = 0.03) and Do you snore? (variable 10) (*p* = 0.02).

The model was rerun with only these significant interaction terms (and main effects for the variables involved) and main effects (Table 3). Significant interactions and effects with coefficients of small absolute value were kept in the model to control for additional factors and improve model fit. The following are the interpretable main effects and significant interactions, along with parameter estimates and *p*-values: Do you snore? (variable 10) (0.68, *p* = 0.01); Physical activity in the past month at visit two and visit 3 (varibles 6:7) (-1.49, *p* = 0.01), Physical activity in the past month at visit 3 with overall hours of sleep per night (variables 7:8) (-0.59, *p* = 0.00), Physical activity in the past month at visit three and napping habit (variables 7:9) (-1.41, *p* = 0.04).

**Table 3:** Parameter estimates and significance for final model

Coefficients:				
	Estimate	Std. Error	z value	Pr(>|z|)
(Intercept)	-2.27	1.54	-1.47	0.1416
**VEG**	-0.12	0.05	-2.19	0.0283
FA	-0.79	0.27	-2.97	0.0029
PAV2	1.02	0.47	2.19	0.0287
PAV3	6.19	1.74	3.55	0.0004
SLPHR	-0.08	0.18	-0.48	0.6329
NAP	1.46	0.59	2.45	0.0144
**SNORE**	0.68	0.26	2.57	0.0101
**FA:SLPHR**	0.10	0.03	3.09	0.0020
**PAV2:PAV3 **	-1.49	0.61	-2.42	0.0157
**PAV3:SLPHR **	-0.59	0.19	-3.03	0.0024
**PAV3:NAP**	-1.41	0.68	-2.08	0.0375

Variable abbreviations: *VEG* Alternative Healthy Eating Index component scores for vegetable intake, *FA* Dehydroepiandrosterone (DHEA) and eicosapentaenoic acid (EPA) fatty acid intake; PAV2(visit 2), PAV3(visit 3) for the question:* “During the past four weeks, did you participate in any physical activities or exercises like running, aerobics, gardening, ball games, or walking for exercise”?; *NAP*: “During a usual week, how many times do you nap for 5 minutes or more;?”*SLPHR:* “How many hours of sleep do you usually get per night (in hours)?”; *SNORE for the question: *“Do you snore?”*

The logistic regression model was then fit to the data with these 15 variables. Because logistic regression is modeling log (p/(1-p)) where p is the probability of the outcome in question (in our case, the negative outcome of HDP), these results can be interpreted as the change in the log odds of a negative outcome for a one unit increase in the effect.

Simply reporting yes to ‘Do you snore?’ at visit one increased the log odds of HDP by 0.68. A higher intake of DHEA and EPA combined with a higher number of hours of reported sleep per night at visit one increased the log odds of HDP by 0.10. An increase in vegetable intake reported at visit one decreased the log odds of HDP by 0.12.

Reporting any physical activity in the past month at both visit two and visit three decreased the log odds of HDP by 1.49. Physical activity in the past month at visit three, combined with a higher number of hours of sleep per night at visit one, decreased the log odds of HDP by 0.59. Reporting yes to napping at least once a week at visit one and reporting any physical activity in the past month at visit three decreased the log odds of HDP by 1.41.

## Discussion

Hypertensive Disorders of Pregnancy cause significant maternal and fetal morbidity and mortality. We hypothesized that lifestyle wellness variables associated with sleep, nutrition, physical activity, resilience, and illness avoidance would impact HDP. Our final analysis of 554 nulliparous women included several findings related to lifestyle wellness variables and HDP risk.

One straightforward finding that indicated an increased risk of HDP in pregnancy was snoring at visit one. There are limited options to screen for hypertension risk in the first trimester and assessing whether a woman snores may be a simple strategy to identify HDP risk early in pregnancy. Research supports that self-reported snoring is related to higher blood pressure in the general population and our results are consistent with emerging research on pregnant populations associating self-reported chronic snoring with adverse pregnancy outcomes like HDP [30, 31]. Known sleep-disordered breathing is associated with HDP, but much of the literature speaks to the risks in later pregnancy when sleep-disordered breathing becomes more pronounced due to the anatomic and physiological changes of later pregnancy [32, 33]. Future research on HDP risk screening should consider snoring prepregnancy and in the first trimester as a variable of interest.

There are limited nutritional guidelines that are specific to pregnancy, although most nutritional guidelines for the general public highlight the importance of vegetables in a healthy diet. Greater vegetable consumption in the three months prior to pregnancy as reported at visit one was associated with decreased risk of HDP in our study. This finding is likely to be associated with the nutrient-dense nature of vegetables and an overall diet habit, established even before pregnancy, that includes more whole foods. Our findings are consistent with previous research that vegetable consumption before and during pregnancy reduces adverse pregnancy outcomes including HDP [34, 35].

One interaction that is difficult to interpret is that increased DHEA and EPA fatty acids in the three months prior to pregnancy combined with more hours of sleep at baseline were associated with HDP. Much research focuses on the health benefits of fish consumption and DHEA and EPA fatty acids for heart health, and some of this research support its reduction of preeclampsia [36, 37]. Typically, adequate sleep is associated with better cardiovascular health outcomes in women [38]. Further study is needed on these variables, their interaction, and their meaning.

Potentially of the greatest importance were the physical activity variables. Any reported physical activity in the four weeks prior to visit two (second trimester) and visit three (later second trimester to early third trimester) reduced the odds of HDP in the sample. This finding is important because it is not based on baseline activity level and includes an area for potential health promotion and intervention development for physical activity during pregnancy to reduce the risk of HDP. In addition, reporting physical activity at visit three with more hours of nightly sleep or with napping habit at visit one reduced risks of HDP. These findings signal that physical activity in later pregnancy is an important variable in HDP risk reduction and may be improved with other health promoting behaviors such as getting recommended sleep, whether through overnight sleep or nap supplementation.

We had hypothesized that resilience traits and avoidance of illness as a representation of preventative health practices would make participants more likely to engage in health promoting wellness lifestyle variables, and further reduce HDP. Our study showed no relationship between these variables and HDP.

## Limitations

Since this study was a secondary analysis, our findings were limited to the data collected in the nuMoM2b dataset. This data was collected during 2010–2013, and since this time HDP research, screening, diagnostics, and management have changed significantly making some of the data potentially irrelevant or less relevant to current day HDP. The sample only included nulliparous, American women who received care at academic medical centers and initiated prenatal care in the first trimester, limiting the generalizability of our findings to other birthing populations. The authors of this study were looking explicitly at select lifestyle and wellness variables and how they may relate to HDP risk, so did not explore in detail or limit for all variables including some relevant comorbidities, such as sleep apnea, which may have further impacted results.

## Conclusion

Hypertension in pregnancy negatively impacts maternal and fetal health. While certain types of HDP, such as preeclampsia, have decreased over the past several decades, chronic hypertension, which has a clear relationship to lifestyle have been on the rise. We hypothesized that wellness variables that often improve chronic diseases may also improve HDP.

Our findings indicate that snoring may be a risk indicator for HDP. Additionally, physical activity in later pregnancy may be an important variable in HDP risk reduction and may further reduce risk when combined with getting recommended sleep. Additionally, simple nutritional changes like increased vegetable consumption may reduce HDP. Our study supports the hypothesis that wellness variables relate to HDP risk reduction and that these variables can be used in interventions to reduce the risk of HDP. These variables should be considered in future research and intervention development to reduce HDP.

These findings should guide research and intervention development. Screening women for snoring and potentially identifying them as at risk may trigger other preventative strategies for HDP, such as aspirin prophylaxis [39]. Simple, low-cost interventions, such as promoting vegetable intake, physical activity and adequate sleep may have a much greater impact on pregnancy health and HDP than previously thought [40].

## Data Availability

Data from the parent nuMoM2b study are currently publicly available through the *Eunice Kennedy Shriver* National Institute of Child Health and Human Development (NICHD) National Institutes of Health Data and Specimen Hub (DASH; https://dash.nichd.nih.gov/).
